# Effects of 2 weeks lower limb immobilization and two separate rehabilitation regimens on gastrocnemius muscle protein turnover signaling and normalization genes

**DOI:** 10.1186/1756-0500-5-166

**Published:** 2012-03-28

**Authors:** Anders Nedergaard, Jakob G Jespersen, Jessica Pingel, Britt Christensen, Nicholas Sroczynski, Henning Langberg, Michael Kjaer, Peter Schjerling

**Affiliations:** 1 Institute of Sports Medicine, Department of Orthopedic Surgery M, Bispebjerg Hospital, Copenhagen, Denmark; 2 Center for Healthy Aging, Faculty of Health Sciences, University of Copenhagen, Copenhagen, Denmark

**Keywords:** Immobilization, Rehabilitation, Resistance training, Akt signaling, Protein supplementation

## Abstract

**Background:**

Limb immobilization causes a rapid loss of muscle mass and strength that requires appropriate rehabilitation to ensure restoration of normal function. Whereas the knowledge of muscle mass signaling with immobilization has increased in recent years, the molecular regulation in the rehabilitation of immobilization-induced muscle atrophy is only sparsely studied. To investigate the phosphorylation and expression of candidate key molecular muscle mass regulators after immobilization and subsequent rehabilitation we performed two separate studies.

**Methods:**

We immobilized the lower limb for 2 weeks followed by the in-house hospital standard physiotherapy rehabilitation (Study 1). Secondly, we conducted an intervention study using the same 2 weeks immobilization protocol during which protein/carbohydrate supplementation was given. This was followed by 6 weeks of rehabilitation in the form of resistance training and continued protein/carbohydrate supplementation (Study 2). We obtained muscle biopsies from the medial gastrocnemius prior to immobilization (PRE), post-immobilization (IMMO) and post-rehabilitation (REHAB) and measured protein expression and phosphorylation of Akt, mTOR, S6k, 4E-BP1, GSK3β, ubiquitin and MURF1 and mRNA expression of Atrogin-1, MURF1, FOXO1, 3 and 4 as well as appropriate housekeeping genes.

**Results:**

In both studies, no changes in protein expression or phosphorylation for any measured protein were observed. In Study 1, FOXO3 and FOXO4 mRNA expression decreased after IMMO and REHAB compared to PRE, whereas other mRNAs remained unchanged. Interestingly, we found significant changes in expression of the putative housekeeping genes GAPDH, HADHA and S26 with immobilization in both studies.

**Conclusions:**

In neither study, the changes in muscle mass associated with immobilization and rehabilitation were accompanied by expected changes in expression of atrophy-related genes or phosphorylation along the Akt axis. Unexpectedly, we observed significant changes in several of the so-called housekeeping genes GAPDH, HADHA and S26 with immobilization in both studies, thereby questioning the usefulness of these genes for normalization of RNA data purposes in muscle immobilization studies.

## Background

Human skeletal muscle loss is a common consequence of physical inactivity, immobilization, aging and disease [1-5]. Two to three weeks of immobilization causes a loss of muscle mass of 5–10%, accompanied by a decrease in strength of 10–20% [6-8], and, accordingly, individuals with injuries that require immobilization of a limb experience a rapid loss of muscle mass and strength. The recovery from such an injury can be complicated by the lag time for strength recovery and is especially true for vulnerable population groups including older and/or frail adults [9].

Effective and quick rehabilitation of muscle mass and especially strength is of key importance to the immobilized individual. Resistance exercise training and adequate nutrition elicit increased muscle mass and strength [10], and combining resistance exercise and essential amino acids plus carbohydrates enhance muscle protein synthesis to a greater degree than either stimulus alone [10,11]. In addition, discrepant results on the effect of amino acid supplementation on muscle loss with immobilization have been reported [12,13].

Despite the importance of appropriate recovery, the rehabilitation of muscle mass and function following immobilization is understudied [14,15] and whereas the knowledge of muscle mass signaling with immobilization has increased in recent years, there is a paucity of studies on the molecular regulation of muscle mass in the rehabilitation of immobilization-induced muscle atrophy [7].

Muscle mass is regulated by the relative rates of protein synthesis and protein breakdown, and the molecular regulation of this includes the key Akt, mammalian target of rapamycin (mTOR), glycogen synthase kinase 3β (GSK3β) and Forkhead box O (FOXO) signaling pathways [16,17]. Akt is activated by insulin and insulin-like growth factor 1 (IGF-1), and the forced transgenic or pharmacologic induction of Akt *in vivo* or *in vitro* is sufficient to cause dramatic muscle hypertrophy and inhibit atrophy [18-22]. Akt affects protein synthesis by allowing assembly of a translation initiation complex through GSK3β and mTOR, of which mTOR activates and inhibits its downstream targets ribosomal protein S6 kinase (S6k) and eukaryotic translation initiation factor 4E binding protein 1 (4E-BP1), respectively. Akt also inhibits FOXO transcription factors, which consist of FOXO1, 3 and 4 in skeletal muscle. The activation of FOXO3 induces muscle loss as well as protein degradation and stimulates the transcription of the ubiquitin ligases Atrogin-1 and Muscle Ring Finger protein 1 (MURF1), which together with FOXO1 belong to a set of muscle atrophy-related genes (“atrogenes”) that are upregulated in several types of murine muscle atrophy [18,23-25].

Accordingly, to investigate the phosphorylation and expression of candidate key molecular muscle mass regulators after immobilization and subsequent rehabilitation, we performed two separate studies. First, we immobilized the lower limb for 2 weeks followed by the in-house hospital standard physiotherapy rehabilitation for another 2 weeks. The aim of the first study was to characterize the effects of the immobilization protocol and standard rehabilitation on muscle signaling and mRNA expression (Study 1). Secondly, we conducted an intervention study using the same 2 weeks immobilization protocol during which protein/carbohydrate supplementation was given. This was followed by 6 weeks of rehabilitation in the form of resistance training and continued protein/carbohydrate supplementation. The aim of the second study was to explore the effects of a resistance training and nutrient supplementation based intervention on muscle signaling and mRNA expression during the recovery from immobilization (Study 2). 6 weeks rehabilitation training was selected in order to aim for full recovery of strength and mass. A protocol of 6 weeks of resistance training rehabilitation after 2 weeks of immobilization has been used previously by others investigating the response of the thigh muscles [7].

For Study 1, we hypothesized that the 2 weeks immobilization would decrease Akt and mTOR signaling along with increased FOXO3, Atrogin-1 and MURF1 mRNA expression, reflecting the loss of muscle mass reported previously for this study [26]. Further, we hypothesized that the standard rehabilitation would be insufficient to recover signaling and mRNA expression relative to post-immobilization. For Study 2, we hypothesized (similar to Study 1) decreased Akt and mTOR signaling along with elevated FOXO3, Atrogin-1 and MURF1 transcripts after immobilization. Regarding the subsequent resistance training and protein/carbohydrate supplement based rehabilitation, we hypothesized a full recovery of mass and strength, reflected by a reversal or normalization to basal levels of signaling and mRNA expression. Of note, as Study 1 and Study 2 are separate studies; no comparisons between the two studies are made.

## Methods

### Study 1: Subjects

8 young men (Table 1) were recruited following online advertising and included in Study 1. As this study also investigated tendon collagen synthesis [26], which is affected by the hormones of contraceptive pills, estradiol and progesterone [27], females were excluded. Eligibility criteria were: Male, 18–30 years of age, no chronic disease, no use of medication, no injuries in the lower body and no obesity (BMI > 30). Incidentally, all were Caucasians and most of the subjects were recreationally active students. All subjects gave, after receiving oral and written information, written, informed consent to participate in the study, in adherence to the declaration of Helsinki. The study was approved by the local Human Subject Ethics Committee of Copenhagen and Frederiksberg [(KF) 11 2006–1743].

**Table 1:** Subject characteristics

Attribute	Unit	Study 1	Study 2
Height	(cm)	184 ± 9	184 ± 7
Weight	(kg)	85 ± 12	84 ± 12
Age	(years)	23 ± 3	24 ± 3
BMI	(kg*m^-2^)	26 ± 3	25 ± 3
Bodyfat	(%)	NR	19.9 ± 5.5
Self-reported PAL-factor		NR	1.78 ± 0.36
Avg. fat intake	(g)	NR	69.3 ± 23.8
Avg. carbohydrate intake	(g)	NR	267.5 ± 58.3
Avg. protein intake	(g)	NR	90.3 ± 30.3
Avg. protein intake	(g/kg)	NR	1.2 ± 0.3

Subject characteristics for Study 1 and Study 2. NR is short for “not recorded”. Data are presented as means ± SD.

### Study 1: Design

**Figure 1:**
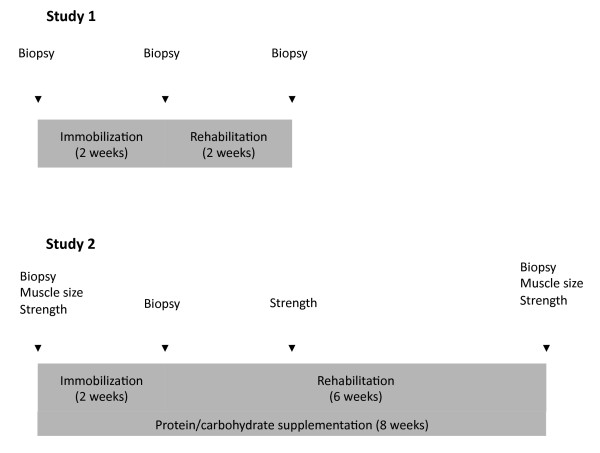
**Study setup.** Study setup and flowchart for Study 1 and 2. Muscle biopsies were only taken from the casted leg. All other measurements were bilateral from casted and control legs.

The subjects were assigned to 2 weeks of immobilizing casting of the lower leg in the non-dominant side, followed by 2 weeks of standard physiotherapy rehabilitation (Figure 1), with the other leg serving as a control. Muscle biopsies were sampled 1 week prior to immobilization (PRE), after the 2-week immobilization period (IMMO) and following 2 weeks of rehabilitation (REHAB (2 W)), and from these biopsies mRNA and protein were isolated for use in RT-qPCR and Western blots. Strength and muscle cross-sectional area (CSA) data from this study have been published previously [26].

### Study 1: Standard rehabilitation protocol

Subjects in Study 1 performed 2 weeks rehabilitation as per standard in-house hospital physiotherapy recommendations at the orthopedic department at Copenhagen University Hospital at Bispebjerg. This protocol was selected in order to characterize the effects of a standard rehabilitation course. This protocol is aimed at mobilizing the joint and reactivating neuromuscular and kinesthetic skills, more so than improving tissue protein turnover or muscle metabolism and strength.

Each of the following exercises were to be performed for approximately 60 s daily: 1) slow flexing/stretching of toes, 2) while seated, lifting a cloth off the floor, gripping it with the toes, 3) while seated, slowly sliding the foot back and forth on a cloth, keeping the heel in contact with the floor, 4) while standing, lifting the toes, 5) while standing, doing calf raises, 6) while standing, doing short lunges, performed until the knee passes the toes, 7) straight knee stretching (targeting the gastrocnemius muscle) and 8) bent-knee stretching (targeting the soleus muscle). The subjects did not maintain exercise logs.

### Study 2: Subjects

In Study 2, another 8 young men (Table 1) were included from similar advertising and recruiting procedures and on the basis of similar eligibility criteria. All subjects gave, after receiving oral and written information, written consent to participate in the study, in adherence to the declaration of Helsinki. The study was approved by the local Human Subject Ethics Committee of Copenhagen and Frederiksberg [(H KF) 319605].

### Study 2: Design

In Study 2, subjects were assigned to an immobilization procedure identical to that used in Study 1, but to another rehabilitation protocol comprised of resistance training for 6 weeks. Moreover, the subjects received protein/carbohydrate supplementation throughout both the immobilization and the rehabilitation period (Figure 1).

Muscle biopsies were sampled 1 week prior to immobilization (PRE), following immobilization (IMMO) and following 6 weeks of rehabilitation (REHAB (6 W)) and muscle size was measured by MRI at the same time points. Muscle strength was measured after 2 (REHAB 2 W) and 6 (REHAB (6 W) weeks of rehabilitation and muscle CSA was measured at the REHAB (6 W) time point as well. Muscle strength and size data from the PRE and IMMO time points in this study have been reported elsewhere [28]. Muscle strength and size were measured as described previously [28,29] Briefly, strength was maximal voluntary (isometric) plantarflexion strength with extended knee exerted against a plate connected to a strain gauge, while muscle CSA was measured from MR slice scans 10 cm distal to the caput fibula.

### Study 2: Rehabilitation exercise protocol

Subjects in Study 2 performed resistance training directed at increasing strength, muscular endurance and increase connective tissue protein turnover [26] three times a week. During weeks 1 and 2, subjects performed 2x15 (set x repetitions) single-legged standing toe raises at bodyweight with full range of motion and 2x15 toe presses in a seated leg press machine at 15–20 repetition maximum (RM). During weeks 3 and 4, subjects performed 3x15 single-legged standing toe raises with additional 10% bodyweight added and 3x15 eccentric toe presses with progressive weight increases (using both legs in the concentric phase, but only one in the eccentric). During weeks 5 and 6, subjects performed the same number of set and repetitions, but with 20% bodyweight added for toe raises. The training was done supervised in-house at the hospital gym and subjects maintained exercise logs that were validated by instructors.

### Study 2: Protein and carbohydrate supplement protocol

Prior to immobilization and during the rehabilitation period, subjects in Study 2 maintained food logs for 3 days, which were used to calculate average habitual nutrient and protein intakes (Table 1).

During the full study (immobilization and rehabilitation), all subjects were instructed to ingest a protein and carbohydrate supplement (20 g of protein and 25 g of dextrose) twice daily and always after exercise. Ingestion of the protein and carbohydrate supplement was monitored only following workouts. The supplement represented a daily addition to protein and energy consumption of 44 and 16%, respectively or an increase in protein consumption of 0.5 ± 0.1 g*kg^−1^*day^−1^. Protein/carbohydrate supplementation began on the first day of immobilization and was continued until the last testing session.

The subjects were administered milk protein: 4 were given whey protein (Lacprodan Whey protein isolate, Arla), and 4 were given alpha-lactalbumin (Lacprodan Alpha-10, Arla; a whey subfraction protein), but as statistical endpoint analysis yielded no difference between these protein supplements at any time, they were pooled for all purposes. These two protein supplements have fairly similar amino acid compositions, e.g. both contain 21% branched-chain amino acids.

### Immobilization

In both studies, the ankle joint and lower part of the non-dominant leg was immobilized in a 90° angle using a cast from below the knee to the toes, leaving the Achilles tendon in a neutral position. The subjects were supplied with crutches and instructed to avoid any load-bearing on the immobilized leg. After the 2 weeks of immobilization, the cast was checked for evidence of weight-bearing (any visible damage or marks after load bearing would exclude the subject), removed, and the subjects were tested and sampled again. Following cast removal, subjects were transported in a wheelchair to the MRI facility and back to the laboratory in order to avoid supporting on the immobilized leg prior to muscle biopsy sampling.

### Muscle biopsy sampling

In both studies, biopsies were sampled from the medial gastrocnemius muscle, using the percutaneous Bergström needle technique [30]. Biopsies were obtained only from the casted leg and not the control leg. Before taking the biopsies, ultrasound imaging was used to identify areas of the muscle with large vessels that should be avoided during the sampling, to prevent unnecessary intramuscular hematomas. Individual biopsy sites were moved 2–3 cm between repeated samplings. In both studies, subjects were sampled at the PRE, IMMO and REHAB time point, with REHAB being after 2 weeks of rehabilitation in Study 1 and after 6 weeks in Study 2.

### RNA isolation

In both studies, approximately 10 mg of muscle tissue was used for RNA isolation. RNA was isolated using phenol extraction [31], using the Trizol kit (Tri-Reagent, Molecular Research). The isolation was performed essentially as proposed, but with two subsequent ethanol precipitation steps, rather than one. The tissue homogenization step was performed using a bead beater (Fastprep-24, MP Biomedicals) shaking specimens in 1 ml Trizol in a 2 ml Biospec tube with 5 stainless steel beads (2.3 mm) and 5 silicon carbide grains for 15 s at speed 4.0 twice. Between shakings samples rested in an ice-water bath. RNA integrity was measured by electrophoresis, running 200 nanograms of RNA on a denaturing agarose gel and visualized with SYBR Green II staining.

### Realtime RT-qPCR

In both studies, the mRNA expression of FOXO4 and the atrophy-related genes FOXO1, FOXO3, Atrogin-1 and MURF1 [23,25] was analyzed by real-time RT-qPCR. Total RNA (500 ng) was converted into cDNA in 20 μl using the OmniScript reverse transcriptase kit (Qiagen, CA, USA) and poly-dT according to the manufacturer’s protocol. For each target, 0.25 μl cDNA was amplified in a 25 μl SYBR Green PCR reaction containing 1X Quantitect SYBR Green Master Mix (Qiagen) and 100 nM of each primer (Table 2). The amplification was monitored real-time using the MX3000P real-time PCR machine (Stratagene). The threshold cycle (Ct) values were related to a standard curve made with the cloned PCR products and specificity ensured by melting curve analyses.

**Table 2:** Antibody and primer specifications

***Primary Antibodies***				
*Epitope*	*Manufacturer*		*Catalog no.*	*Dilution*
Akt	Cell Signaling		2920	1:2,000
p-Akt (T308)	Cell Signaling		2965	1:2,000
p-Akt (S473)	Cell Signaling		4060	1:2,000
mTOR	Cell Signaling		2983	1:2,000
p-mTOR (S2448)	Cell Signaling		2971	1:2,000
p-mTOR (S2481)	Cell Signaling		2974	1:2,000
S6K	Cell Signaling		9202	1:2,000
p-S6K (T389)	Cell Signaling		9206	1:2,000
GSK3β	Abcam		Ab31826	1:2,000
P-GSK3β (S9)	Cell Signaling		9336	1:2,000
4E-BP1	Santa Cruz		Sc-81149	1:200
p-4E-BP1 (T37/46)	Cell Signaling		2855	1:2,000
MURF1	Abcam		Ab4125	1:2,000
***Secondary antibodies***				
*Epitope*	*Fluorophore conjugate*	*Manufacturer*	*Catalog no.*	*Dilution*
Mouse Ig	Alexa 680	Invitrogen	A21057	1:10,000
Goat Ig	Alexa 680	Invitrogen	A21084	1:10,000
Rabbit Ig	Dylight 800	Pierce	35571	1:10,000
***PCR primers***				
*Target*	*Primers*			
FOXO1	Sense	GCCCAACCAAAGCTTCCCACAC		
	Antisense	TGGACTGCTTCTCTCAGTTCCTGCT		
FOXO3	Sense	GCTGGGTGCCAGGCTGAAGG		
	Antisense	TTGGCAAAGGGTTTTCTCTGTAGGT		
FOXO4	Sense	GATGAGGGCGAGGGACTGGA		
	Antisense	TCCACATCTGAAGCAGGGGACA		
MURF1	Sense	TGGGGGAGCCACCTTCCTCT		
	Antisense	ATGTTCTCAAAGCCCTGCTCTGTCT		
Atrogin-1	Sense	TGTTACCCAAGGAAAGAGCAGTATGGA		
	Antisense	ACGGAGCAGCTCTCTGGGTTATTG		
HADHA	Sense	GCGAGTCTGAAGCTGCCTCCTAA		
	Antisense	GGCACATGACTGCCTCATTCACA		
S26	Sense	AACACCCCCACCCCGATTTAGAC		
	Antisense	GAACTCAGCTCCTTACATGGGCTTT		
Cyclophillin	Sense	TGCAGACAAGGTCCCAAAGACAG		
	Antisense	TGAAAGCAGGAACCCTTATAACCA		
β2-microglobulin	Sense	GCTGTGCTCGCGCTACTCTCTCT		
	Antisense	TCTGCTGGATGACGTGAGTAAACCT		
GAPDH	Sense	CCTCCTGCACCACCAACTGCTT		
	Antisense	GAGGGGCCATCCACAGTCTTCT		

Specification of antibodies used for Western blots and primers used for qPCR.

In the initial PCR assay we measured Glyceraldehyde 3-phosphate dehydogenase (GAPDH) and Ribosomal protein, large, P0 (RPLP0) expression for normalization purposes, but as their expression changed in relation to each other, we proceeded to measure more putative housekeeping genes (26S, β2-microglobulin, Cyclophillin A and HADHA) in another assay. Therefore, we measured expression of six putatively stably expressed “housekeeping” genes GAPDH, RPLP0, β2-microglobulin (β2MG), Cyclophillin A, hydroxyacyl-coenzyme A dehydrogenase alpha subunit (HADHA) and ribosomal protein S26 (S26) and did indeed find significant variation within these supposedly stably expressed genes (See Results section). Thus, we proceeded to use the “GeNorm” algorithm (VBA applet for Excel) [32] to identify the housekeeping genes most stably expressed. The GeNorm housekeeping gene analysis revealed the most variable genes to be GAPDH, S26 and HADHA in descending order of variability. Including β2MG, RPLP0 and Cyclophillin A in the geometric mean used for normalization yielded a variability score of 0.151, complying with the “stability threshold” score of 0.150 recommended in Vandesompele et al. [32]. We proceeded to generate geometric means of the expression of these genes and used the resulting figure for normalization. Statistics were done on normalized and log-transformed numbers. Finally, we back-transformed means and SEM’s for reporting and graphical visualization.

### Protein isolation

Approximately 10 mg of muscle tissue was homogenized as described for RNA isolation, but performed in 200 μl homogenization buffer (50 mM Tris–HCl, 1 mM EDTA, 1 mM EGTA, 10 mM β-glycerophosphate. 50 mM NaF, 0.5 mM sodium orthovanadate, 0.1% 2-mercaptoethanol, 0.1% Triton-X and protease inhibitor (Complete, Roche), adjusted to pH 7.5). Following bead beating, samples were briefly spun down and aliquots of the resulting supernatant were used for protein concentration determination, using the EZQ protein quantitation kit (Molecular Probes) and CCD camera (Kodak Image Station 3000MMpro). Prior to loading, aliquots of the samples were diluted to a final concentration of 1 ug/ul, using 4X Laemmli buffer, to a final concentration of 1X Laemmli.

### Western blots

In both studies, we measured total protein and phosphorylation for Akt (T308 and S473), mTOR (S2448 and S2481), S6k (T389), 4EBP1 (T37/46) and GSK3β (S9) as well as protein expression of ubiquitin and MURF1 (Table 2). Western blotting was performed essentially as previously described [33]. For electrophoresis, 20 μg protein per well was loaded on Criterion 4–12% gradient gels (Bio-Rad) and run at 200 V for 1 h. The gels were cut into pieces corresponding to target sizes. Gel slabs containing proteins larger than 110 kDa were blotted in absence of methanol, while the remaining gel pieces were blotted in the presence of methanol. Gels were blotted (Trans-blot cell, Bio-Rad, 400 mA, 2 h) to polyvinylidene difluoride membranes (Amersham Hybond LFP, GE Healthcare) in transfer buffer (50 mM Tris-base, 383 mM glycine, 20% v/v methanol), washed briefly in distilled water and blocked for 30 min in 10% Odyssey Blocking buffer in phosphate-buffered saline (PBS). Following blocking, membranes were washed in Tris-buffered saline with Tween-20 (TBST) (50 mM pH 7.4 Tris, 150 mM NaCl, 0.1% Tween-20, pH 7.4) and incubated with primary antibodies (Table 2) overnight. Following primary antibody incubation, membranes were washed in TBST and incubated with appropriate secondary antibodies conjugated to Dylight 800 or Alexa 680 fluorophores (Table 2). For visualization, blots were scanned using an Odyssey scanner (LiCor) at 84 μm resolution and standard settings and quantified using ImageJ (National Institutes of Health, USA). Where appropriate, total and phospho-antibodies were incubated simultaneously on the same membrane, each in its own wavelength channel. Prior to statistical analysis, results were normalized to individual PRE values and log-transformed. Finally, we back-transformed means and SEM’s for reporting and graphical visualization.

### Statistics

No statistical comparisons were made between Study 1 and 2, as we consider them separate studies. Statistical analysis was, however, performed in an identical manner between the two studies.

For Study 2 only, muscle size (CSA) and strength (MVC) PRE-normalized data were subjected to repeated measures one-way ANOVA (and post hoc testing) individually for each leg.

Western blot and RT-qPCR data were normalized as described in their respective paragraphs and log-transformed prior to being subjected to repeated measures one-way ANOVA (and post hoc testing). The log-transformation was used to ensure that the data approached normal distribution as closely as possible.

In figures, all data are presented as means ± SEM (for RT-qPCR and Western blot data back-transformed means ± SEM). In Table 1, data are presented as means ± SD. CSA, MVC and mRNA analyses were done using Sigmastat 3.5.0.54 (Systat Software, 2006) with Student-Newman-Keuls correction for multiple testing, whereas protein data were analyzed using SAS 9.1.3 (SAS Institute 2008). For all post hoc tests we applied a significance threshold of 0.05.

## Results

### Study 1

#### mRNA expression

**Figure 2:**
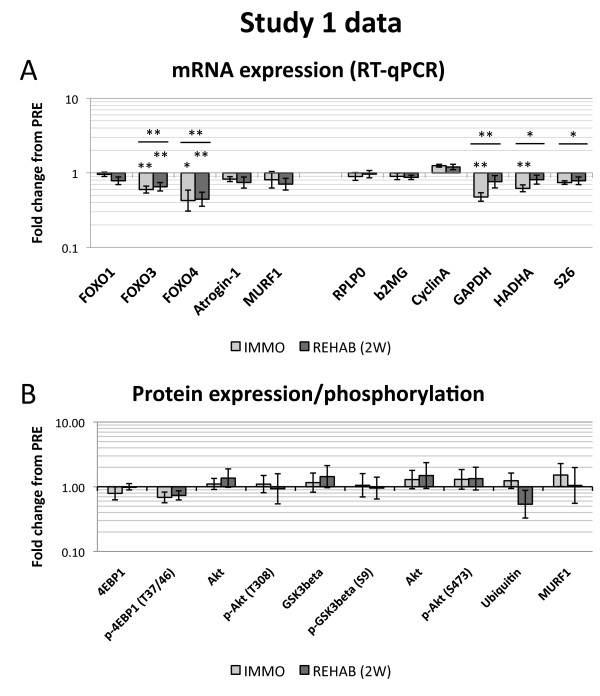
**Data for Study 1. A:** mRNA expression measured by RT-qPCR. mRNA data are normalized to geometric means of Cyclophillin A, β2-microglobulin and RPLP0 and are relative to individual PRE values. Target genes are presented in the left side panel, whereas putative housekeeping genes are presented in the right side panel. Data are presented as back-transformed mean ± SEM. Light gray columns represent IMMO and dark gray REHAB. **B:** Total protein and protein phosphorylation measured by Western blot, relative to individual PRE values. Data are presented as back-transformed mean ± SEM. Total Akt is represented twice as it was measured along with each phospho-Akt. Light gray columns represent IMMO and dark gray REHAB. * denotes a difference from PRE with 0.05 > *p* ≥ 0.01. ** denotes a difference from PRE with 0.01 > *p* ≥ 0.001. Underscored asterisks denote main (time) effects.

As for mRNA (Figure 2A), we observed significant time effects for FOXO3 (*p* = 0.004), FOXO4 (*p* = 0.013), GAPDH (*p* = 0.005), HADHA (*p* = 0.010) and S26 (*p* = 0.037) transcripts. For FOXO3 and FOXO4 this was manifested in the form of a downregulation at the IMMO time point (−40% for FOXO3 (*p* = 0.006) and −58% for FOXO4 (*p* = 0.026)) that persisted until the REHAB (2 W) time point (−19% for FOXO3 (*p* = 0.006) and −35% for FOXO4 (*p* = 0.009)). GAPDH and HADHA were also downregulated at the IMMO time point (−53% for GAPDH (*p* = 0.004) and −40% for HADHA (*p* = 0.008)), but returned almost back to baseline expression at the REHAB time point. Despite manifesting a time effect, S26 was not significantly downregulated at either time point (Figure 2A).

#### Protein expression and phosphorylation

Despite the obvious changes in muscle mass [26], we observed no significant modulation of total or phosphoprotein levels of Akt, GSK3β, 4EBP1, ubiquitin or MURF1 in Study 1 (Figure 2B). Total and phosphorylated levels of mTOR and S6k were below the detection threshold.

### Study 2

#### Muscle strength and size

In Study 2, we report additional muscle strength and size data for the REHAB time points. For strength, no significant time effects were detected in either leg. Following 2 and 6 weeks of rehabilitation, strength was elevated to levels slightly higher than those recorded before immobilization, but these differences did not reach significance. For muscle size, no differences were observed between the REHAB (6 W) time point and PRE.

#### mRNA expression

**Figure 3:**
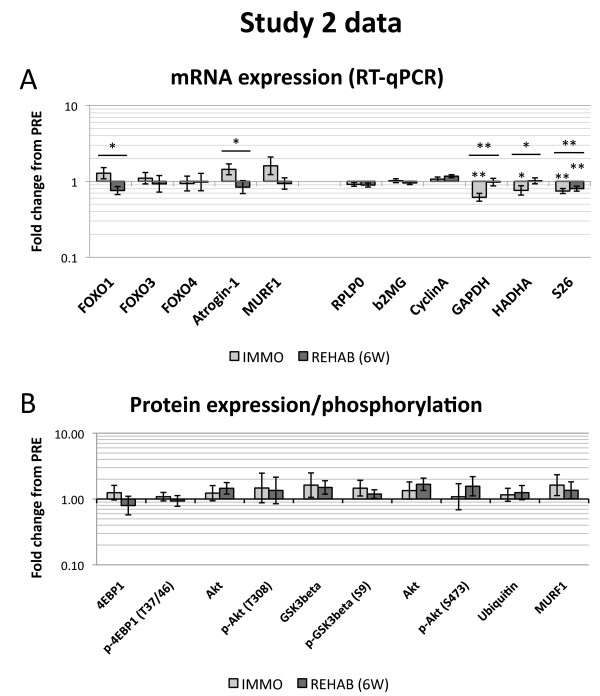
**Data for Study 2. A:** mRNA expression measured by RT-qPCR. mRNA data are normalized to geometric means of Cyclophillin A, β2-microglobulin and RPLP0 and are relative to individual PRE values. Target genes are presented in the left side panel, whereas putative housekeeping genes are presented in the right side panel. Data are presented as back-transformed mean ± SEM. Light gray columns represent IMMO and dark gray REHAB. **B:** Total protein and protein phosphorylation measured by Western blot, relative to individual PRE values. Data are presented as back-transformed mean ± SEM. Total Akt is represented twice as it was measured along with each phospho-Akt. Light gray columns represent IMMO and dark gray REHAB. * denotes a difference from PRE with 0.05 > *p* ≥ 0.01. ** denotes a difference from PRE with 0.01 > *p* ≥ 0.001. Underscored asterisks denote main (time) effects.

For mRNA (Figure 3A), we observed time effects for FOXO1 (*p* = 0.030), Atrogin-1 (*p* = 0.046), GAPDH (*p* = 0.001), HADHA (*p* = 0.035) and S26 (*p* = 0.001). Notably, for neither FOXO1 nor Atrogin-1, the main effect could be observed as deviations from PRE. For both GAPDH and HADHA, we observed a downregulation at the IMMO time point (−39% for GAPDH (*p* = 0.001) and −24% for HADHA (*p* = 0.043)) and a subsequent return to baseline expression, whereas for S26 we found a downregulation at the IMMO time point that persisted throughout the REHAB (6 W) time point (−26% at IMMO (*p* = 0.002) and −20% at REHAB (*p* = 0.006) (Figure 3A).

#### Protein expression and phosphorylation

Contrary to the changes in muscle mass reported previously [28], we found no significant modulation of total or phosphoprotein levels of Akt, GSK3β, 4EBP1, ubiquitin or MURF1 in Study 2 (Figure 3B). Total and phosphorylated levels of mTOR and S6k were below the detection threshold.

## Discussion

For Study 1, we hypothesized that the 2 weeks immobilization would decrease Akt and mTOR signaling along with increased FOXO3, Atrogin-1 and MURF1 mRNA expression, reflecting the loss of muscle mass previously reported for this study [26]. We observed no changes in Akt and mTOR signaling, and of FOXO3, Atrogin-1 and MURF1 only FOXO3 was significantly downregulated after immobilization, which is opposite of what we expected. Furthermore, we hypothesized that the standard rehabilitation would be insufficient to recover signaling and mRNA expression relative to post-immobilization. As hypothesized, signaling and all mRNAs, except the downregulated FOXO3, were unchanged with rehabilitation relative to the IMMO time point.

For Study 2, we hypothesized (similar to study 1) decreased Akt and mTOR signaling along with elevated FOXO3, Atrogin-1 and MURF1 transcripts after immobilization. Unexpectedly, Akt and mTOR signaling and the measured mRNAs remained unchanged after immobilization. Regarding the subsequent combined resistance training and protein/carbohydrate supplementation based rehabilitation, we hypothesized a full recovery of mass and strength, reflected by a reversal or normalization to basal levels of signaling and mRNA expression. Indeed, this rehabilitation protocol restored both mass and strength, whereas signaling and mRNA expression remained unchanged after rehabilitation relative to post-immobilization.

### Protein expression and phosphorylation

To our knowledge, we present the first results on Akt, 4E-BP1 and GSK3β phosphorylation following rehabilitation after immobilization, and, unexpectedly, we show no changes. Contrary to our hypothesis for both Study 1 and Study 2, the total and phosphorylation levels of the measured proteins remained unchanged after 2 weeks immobilization. This is similar to a previous human 10 days immobilization study reporting unchanged total and phosphorylated levels of Akt, S6k and 4E-BP1 [6], although a decrease in Akt S473 phosphorylation after 20 days immobilization has been observed as well [34]. Of note, these studies [6,34] were performed on vastus lateralis muscle, whereas our data is generated from gastrocnemius muscle, which may affect the response. Moreover, the high variation in the data may explain, at least partly, the absence of significant differences with time or treatment (Figures 2B and 3B). Due to this variation we cannot exclude the possibility that some minor modulation occurs, but it is unlikely that the observed variation masks large changes in phosphorylation status.

### mRNA expression

Unexpectedly, we observed a significant decrease in FOXO3 and FOXO4 mRNA expression with immobilization as well as rehabilitation in Study 1 (Figure 2A). This could be interpreted as an anti-catabolic response, but given that the subjects were atrophic at the IMMO time point [26] and anabolic at the REHAB time point, this represent a discrepancy between the suggested gene function and the physiological condition. We cannot rule out the possibility that this response is secondary to other regulation events and thus not part of a primary response. It is still a matter of debate what the individual biological contributions of each FOXO gene are and under what circumstances they are activated, but murine *in vitro* and *in vivo* studies indicate that the FOXO genes should be considered negative regulators of muscle mass [20,35-37].

FOXO transcript behavior *in vivo* is poorly characterized with immobilization and our data represent novel findings on the effects of immobilization and rehabilitation on FOXO transcript regulation in human muscle. Regarding the sparse literature that does exist, FOXO3 reporter activity has been shown to be upregulated with immobilization in an animal model [38], whereas in a human model, 3 and 20 days of unloading produced no changes in FOXO1 or FOXO3 mRNA and protein expression levels [34,39].

As for the “atrogenes” Atrogin-1 and MURF1 [18,23], in Study 2, we observed a tendency (*p* = 0.10) towards an increase from PRE in Atrogin-1 expression (Figure 3A) after immobilization. Both Atrogin-1 and MURF1 have previously been shown to be upregulated in a 14-day human immobilization study [7], although in that study, the upregulation of MURF1 did not reach statistical significance. Other time course studies show that Atrogin-1 and/or MURF1 expression increases in the initial phase of immobilization and gradually declines, sometimes even to levels below baseline [6,40]. Also, in the study by Jones et al. [7], Atrogin-1 and MURF1 seem to return to pre-levels with rehabilitation like in the present study, but with an apparent “undershoot”, a finding we cannot replicate due to our less detailed time course.

### Normalization of mRNA expression

In the initial PCR assay we measured GAPDH and RPLP0 expression for normalization purposes, but as their expression changed in relation to each other, we proceeded to measure more putative housekeeping genes (26S, β2-microglobulin, Cyclophillin A and HADHA) in another assay. Actually, we have previously shown that our most frequently used normalization genes RPLP0 and GAPDH change in rats with hindlimb unloading, relative to muscle weight as well as relative to total RNA [41]. Accordingly, we used the method suggested by Vandesompele et al. [32] to determine which genes were the most stably expressed and used those for normalization. This analysis indicated GAPDH, HADHA and S26 to be the most variable of the measured housekeeping genes. Indeed, in both of our immobilization experiments, all of these genes displayed decreases in expression with immobilization, with GAPDH actually returning to baseline levels after rehabilitation (Figures 2A and 3A), thereby making these genes highly unsuitable for normalization purposes in immobilization studies. This is particularly relevant for GAPDH, being one of the most common normalization genes used and due to it responding drastically to immobilization, thereby not being fit for normalization purposes. This may have implications for interpretation of existing immobilization studies using GAPDH as an internal reference in RT-qPCR analyses without further validation of housekeeping gene expression stability [42-44], and should most certainly be kept in mind in future immobilization studies measuring mRNA expression.

### Limitations

The discontinuous nature of these studies represents a major limitation. The missing time points and discrepant time courses do not allow for interstudy comparison, which would otherwise have been informative. Moreover, using the contralateral limb as a control in immobilization studies is controversial as the contralateral limb may experience a compensatory training effect due to increased stress [45]. This is especially true when combined with protein/carbohydrate supplementation as this is thought to contribute to hypertrophy only in conjunction with exercise or increased workload [46]. Therefore, the contralateral leg cannot be considered a true, passive control within this study.

The variation in our Western blots was higher than expected; part of this can likely be ascribed to keeping our stock samples in a native buffer. Since then, we have found that denaturing samples immediately following sample homogenization yields more consistent results.

In Study 1, no exercise logs were maintained, and this could mask low compliance, which in turn could cause impaired rehabilitation efficiency. Moreover, it must be noted that the ingestion of supplements were only supervised on post-workout occasions, allowing for compliance issues.

## Conclusions

In Study 2, we observed full strength recovery after 2 weeks of rehabilitation. In both studies, no changes in protein expression or phosphorylation for any measured protein were observed. In Study 1, FOXO3 and FOXO4 mRNA expression decreased after IMMO and REHAB compared to PRE, whereas other mRNAs remained unchanged. Interestingly, we found significant changes in expression of the putative housekeeping genes GAPDH, HADHA and S26 with immobilization in both studies.

Accordingly, in either study, changes in muscle mass after immobilization and subsequent rehabilitation were not reflected in total protein or phosphorylation status of Akt and mTOR pathway components. The changes in the so-called housekeeping genes GAPDH, HADHA and S26 with immobilization in both studies question the usefulness of these genes for normalization purposes in muscle immobilization studies, and a more thorough characterization of changes in housekeeping genes is of relevance to future immobilization studies.

In perspective, this calls for further research, preferably a full randomized controlled trial, comparing the contributions of protein/carbohydrate supplementation and resistance training, individually as well as in combination, on immobilization-induced atrophy and rehabilitation-induced hypertrophy and related molecular regulation.

## Competing interests

The authors declare that they have no competing interests.

## Authors’ contributions

AN contributed to biopsy sampling, RNA and protein isolation, RT-qPCR and Western blot analyses, statistics, data analysis and interpretation, and manuscript writing. JGJ contributed to biopsy sampling, RNA and protein isolation, RT-qPCR, Western blotting, data interpretation and manuscript writing. JP contributed to study design, recruiting and training in Study 2 and part of Study 1 as well as strength and muscle CSA measurements. BC contributed to study design, recruiting and training in Study 1 as well as strength and CSA measurements. NS contributed to RNA isolation and mRNA quantification. HL contributed to study concept and design, organizing and funding both trials. MK contributed to study concept, design and funding and to manuscript writing. PS was providing technical support and guidance for laboratory work, contributed to data analysis, statistics and interpretation and contributed to manuscript writing. All authors read and approved the final manuscript.

## References

[B1] Olsen RH Krogh-Madsen R Thomsen C Booth FW Pedersen BK Metabolic responses to reduced daily steps in healthy nonexercising men JAMA 2008 299 1261 1263 18349087 10.1001/jama.299.11.1259

[B2] Jespersen JG Nedergaard A Andersen LL Schjerling P Andersen JL Myostatin expression during human muscle hypertrophy and subsequent atrophy: increased myostatin with detraining Scand J Med Sci Sports 2011 21 215 23 10.1111/j.1600-0838.2009.01044.x 19903317

[B3] Phillips SM Glover EI Rennie MJ Alterations of protein turnover underlying disuse atrophy in human skeletal muscle J Appl Physiol 2009 107 645 654 19608931 10.1152/japplphysiol.00452.2009

[B4] Baumgartner RN Koehler KM Gallagher D Romero L Heymsfield SB Ross RR Garry PJ Lindeman RD Epidemiology of sarcopenia among the elderly in New Mexico Am J Epidemiol 1998 147 755 763 10.1093/oxfordjournals.aje.a009520 9554417

[B5] Evans WJ Morley JE Argiles J Bales C Baracos V Guttridge D Jatoi A Kalantar-Zadeh K Lochs H Mantovani G Cachexia: a new definition Clin Nutr 2008 27 793 799 10.1016/j.clnu.2008.06.013 18718696

[B6] de Boer MD Selby A Atherton P Smith K Seynnes OR Maganaris CN Maffulli N Movin T Narici MV Rennie MJ The temporal responses of protein synthesis, gene expression and cell signalling in human quadriceps muscle and patellar tendon to disuse J Physiol 2007 585 241 251 10.1113/jphysiol.2007.142828 17901116 PMC2375459

[B7] Jones SW Hill RJ Krasney PA O’Conner B Peirce N Greenhaff PL Disuse atrophy and exercise rehabilitation in humans profoundly affects the expression of genes associated with the regulation of skeletal muscle mass FASEB J 2004 18 1025 1027 15084522 10.1096/fj.03-1228fje

[B8] Glover EI Phillips SM Oates BR Tang JE Tarnopolsky MA Selby A Smith K Rennie MJ Immobilization induces anabolic resistance in human myofibrillar protein synthesis with low and high dose amino acid infusion J Physiol 2008 586 6049 6061 10.1113/jphysiol.2008.160333 18955382 PMC2655417

[B9] Hvid L Aagaard P Justesen L Bayer ML Andersen JL Ortenblad N Kjaer M Suetta C Effects of aging on muscle mechanical function and muscle fiber morphology during short-term immobilization and subsequent retraining J Appl Physiol 2010 109 1628 1634 10.1152/japplphysiol.00637.2010 20864557

[B10] Dreyer HC Drummond MJ Pennings B Fujita S Glynn EL Chinkes DL Dhanani S Volpi E Rasmussen BB Leucine-enriched essential amino acid and carbohydrate ingestion following resistance exercise enhances mTOR signaling and protein synthesis in human muscle Am J Physiol Endocrinol Metab 2008 294 E392 E400 18056791 10.1152/ajpendo.00582.2007 PMC2706121

[B11] Fujita S Dreyer HC Drummond MJ Glynn EL Cadenas JG Yoshizawa F Volpi E Rasmussen BB Nutrient signalling in the regulation of human muscle protein synthesis J Physiol 2007 582 813 823 10.1113/jphysiol.2007.134593 17478528 PMC2075348

[B12] Paddon-Jones D Sheffield-Moore M Urban RJ Sanford AP Aarsland A Wolfe RR Ferrando AA Essential amino acid and carbohydrate supplementation ameliorates muscle protein loss in humans during 28 days bedrest J Clin Endocrinol Metab 2004 89 4351 4358 10.1210/jc.2003-032159 15356032

[B13] Trappe TA Burd NA Louis ES Lee GA Trappe SW Influence of concurrent exercise or nutrition countermeasures on thigh and calf muscle size and function during 60 days of bed rest in women Acta Physiol (Oxf) 2007 191 147 159 10.1111/j.1748-1716.2007.01728.x 17655736

[B14] Marimuthu K Murton AJ Greenhaff PL Mechanisms regulating muscle mass during disuse atrophy and rehabilitation in humans J Appl Physiol 2011 110 555 560 10.1152/japplphysiol.00962.2010 21030670

[B15] Murton AJ Greenhaff PL Physiological control of muscle mass in humans during resistance exercise, disuse and rehabilitation Curr Opin Clin Nutr Metab Care 2010 13 249 254 10.1097/MCO.0b013e3283374d19 20110809

[B16] Sandri M Signaling in muscle atrophy and hypertrophy Physiology (Bethesda) 2008 23 160 170 10.1152/physiol.00041.2007 18556469

[B17] Ruegg MA Glass DJ Molecular mechanisms and treatment options for muscle wasting diseases Annu Rev Pharmacol Toxicol 2011 51 373 395 10.1146/annurev-pharmtox-010510-100537 20936944

[B18] Bodine SC Latres E Baumhueter S Lai VK Nunez L Clarke BA Poueymirou WT Panaro FJ Na E Dharmarajan K Identification of ubiquitin ligases required for skeletal muscle atrophy Science 2001 294 1704 1708 10.1126/science.1065874 11679633

[B19] Bodine SC Stitt TN Gonzalez M Kline WO Stover GL Bauerlein R Zlotchenko E Scrimgeour A Lawrence JC Glass DJ Yancopoulos GD Akt/mTOR pathway is a crucial regulator of skeletal muscle hypertrophy and can prevent muscle atrophy in vivo Nat Cell Biol 2001 3 1014 1019 10.1038/ncb1101-1014 11715023

[B20] Zhao J Brault JJ Schild A Cao P Sandri M Schiaffino S Lecker SH Goldberg AL FoxO3 coordinately activates protein degradation by the autophagic/lysosomal and proteasomal pathways in atrophying muscle cells Cell Metab 2007 6 472 483 10.1016/j.cmet.2007.11.004 18054316

[B21] Rommel C Bodine SC Clarke BA Rossman R Nunez L Stitt TN Yancopoulos GD Glass DJ Mediation of IGF-1-induced skeletal myotube hypertrophy by PI(3)K/Akt/mTOR and PI(3)K/Akt/GSK3 pathways Nat Cell Biol 2001 3 1009 1013 10.1038/ncb1101-1009 11715022

[B22] Lai KM Gonzalez M Poueymirou WT Kline WO Na E Zlotchenko E Stitt TN Economides AN Yancopoulos GD Glass DJ Conditional activation of akt in adult skeletal muscle induces rapid hypertrophy Mol Cell Biol 2004 24 9295 9304 10.1128/MCB.24.21.9295-9304.2004 15485899 PMC522257

[B23] Sandri M Sandri C Gilbert A Skurk C Calabria E Picard A Walsh K Schiaffino S Lecker SH Goldberg AL Foxo transcription factors induce the atrophy-related ubiquitin ligase atrogin-1 and cause skeletal muscle atrophy Cell 2004 117 399 412 10.1016/S0092-8674(04)00400-3 15109499 PMC3619734

[B24] Sacheck JM Hyatt JP Raffaello A Jagoe RT Roy RR Edgerton VR Lecker SH Goldberg AL Rapid disuse and denervation atrophy involve transcriptional changes similar to those of muscle wasting during systemic diseases FASEB J 2007 21 140 155 17116744 10.1096/fj.06-6604com

[B25] Lecker SH Jagoe RT Gilbert A Gomes M Baracos V Bailey J Price SR Mitch WE Goldberg AL Multiple types of skeletal muscle atrophy involve a common program of changes in gene expression FASEB J 2004 18 39 51 10.1096/fj.03-0610com 14718385

[B26] Christensen B Dyrberg E Aagaard P Kjaer M Langberg H Short-term immobilization and recovery affect skeletal muscle but not collagen tissue turnover in humans J Appl Physiol 2008 105 1845 1851 10.1152/japplphysiol.90445.2008 18927270

[B27] Hansen M Kongsgaard M Holm L Skovgaard D Magnusson SP Qvortrup K Larsen JO Aagaard P Dahl M Serup A Effect of estrogen on tendon collagen synthesis, tendon structural characteristics, and biomechanical properties in postmenopausal women J Appl Physiol 2009 106 1385 1393 10.1152/japplphysiol.90935.2008 18927264

[B28] Pingel J Moerch L Kjaer M Langberg H The influence of training status on the drop in muscle strength after acute exercise Eur J Appl Physiol 2009 106 605 611 10.1007/s00421-009-1055-0 19363682

[B29] Magnusson SP Aagaard P Dyhre-Poulsen P Kjaer M Load–displacement properties of the human triceps surae aponeurosis in vivo J Physiol 2001 531 277 288 10.1111/j.1469-7793.2001.0277j.x 11179410 PMC2278435

[B30] Bergstrom J Percutaneous needle biopsy of skeletal muscle in physiological and clinical research Scand J Clin Lab Invest 1975 35 609 616 10.3109/00365517509095787 1108172

[B31] Chomczynski P Sacchi N Single-step method of RNA isolation by acid guanidinium thiocyanate-phenol-chloroform extraction Anal Biochem 1987 162 156 159 2440339 10.1006/abio.1987.9999

[B32] Vandesompele J De Preter K Pattyn F Poppe B Van Roy N De Paepe A Speleman F Accurate normalization of real-time quantitative RT-PCR data by geometric averaging of multiple internal control genes Genome Biol 2002 3 RESEARCH0034 12184808 10.1186/gb-2002-3-7-research0034 PMC126239

[B33] Jespersen JG Nedergaard A Reitelseder S Mikkelsen UR Dideriksen KJ Agergaard J Kreiner F Pott FC Schjerling P Kjaer M Activated protein synthesis and suppressed protein breakdown signaling in skeletal muscle of critically ill patients PLoS One 2011 6 e18090 10.1371/journal.pone.0018090 21483870 PMC3069050

[B34] Sakuma K Watanabe K Hotta N Koike T Ishida K Katayama K Akima H The adaptive responses in several mediators linked with hypertrophy and atrophy of skeletal muscle after lower limb unloading in humans Acta Physiol (Oxf) 2009 197 151 159 10.1111/j.1748-1716.2009.01995.x 19432591

[B35] Mammucari C Milan G Romanello V Masiero E Rudolf R Del Piccolo P Burden SJ Di Lisi R Sandri C Zhao J FoxO3 controls autophagy in skeletal muscle in vivo Cell Metab 2007 6 458 471 10.1016/j.cmet.2007.11.001 18054315

[B36] McLoughlin TJ Smith SM DeLong AD Wang H Unterman TG Esser KA FoxO1 induces apoptosis in skeletal myotubes in a DNA-binding-dependent manner Am J Physiol Cell Physiol 2009 297 C548 C555 10.1152/ajpcell.00502.2008 19553561 PMC2740395

[B37] Moylan JS Smith JD Chambers MA McLoughlin TJ Reid MB TNF induction of atrogin-1/MAFbx mRNA depends on Foxo4 expression but not AKT-Foxo1/3 signaling Am J Physiol Cell Physiol 2008 295 C986 C993 10.1152/ajpcell.00041.2008 18701653 PMC2575831

[B38] Dodd SL Gagnon BJ Senf SM Hain BA Judge AR Ros-mediated activation of NF-kappaB and Foxo during muscle disuse Muscle Nerve 2010 41 110 113 10.1002/mus.21526 19813194 PMC2796694

[B39] Gustafsson T Osterlund T Flanagan JN von Walden F Trappe TA Linnehan RM Tesch PA Effects of 3 days unloading on molecular regulators of muscle size in humans J Appl Physiol 2010 109 721 727 20538844 10.1152/japplphysiol.00110.2009

[B40] Abadi A Glover EI Isfort RJ Raha S Safdar A Yasuda N Kaczor JJ Melov S Hubbard A Qu X Limb immobilization induces a coordinate down-regulation of mitochondrial and other metabolic pathways in men and women PLoS One 2009 4 e6518 10.1371/journal.pone.0006518 19654872 PMC2716517

[B41] Heinemeier KM Olesen JL Haddad F Schjerling P Baldwin KM Kjaer M Effect of unloading followed by reloading on expression of collagen and related growth factors in rat tendon and muscle J Appl Physiol 2009 106 178 186 18988763 10.1152/japplphysiol.91092.2008

[B42] Urso ML Scrimgeour AG Chen YW Thompson PD Clarkson PM Analysis of human skeletal muscle after 48 h immobilization reveals alterations in mRNA and protein for extracellular matrix components J Appl Physiol 2006 101 1136 1148 10.1152/japplphysiol.00180.2006 16763108

[B43] Wagatsuma A Yamazaki Y Mizuno K Yamada S Molecular properties and gene expression of albumin in the skeletal muscle following hindlimb immobilization in a shortened position Acta Neuropathol 2001 101 540 546 11515781 10.1007/s004010000311

[B44] Hortobagyi T Dempsey L Fraser D Zheng D Hamilton G Lambert J Dohm L Changes in muscle strength, muscle fibre size and myofibrillar gene expression after immobilization and retraining in humans J Physiol 2000 524 Pt 1 293 304 10747199 10.1111/j.1469-7793.2000.00293.x PMC2269843

[B45] Lu D-X Käser L Muntener M Experimental changes to limb muscles elicit contralateral reactions: the problem of controls J Exp Biol 1999 202 1691 1700 10333514 10.1242/jeb.202.12.1691

[B46] Rennie MJ Wackerhage H Spangenburg EE Booth FW Control of the size of the human muscle mass Annu Rev Physiol 2004 66 799 828 10.1146/annurev.physiol.66.052102.134444 14977422

